# Insulin Confers Differing Effects on Neurite Outgrowth in Separate Populations of Cultured Dorsal Root Ganglion Neurons: The Role of the Insulin Receptor

**DOI:** 10.3389/fnins.2018.00732

**Published:** 2018-10-10

**Authors:** Bence András Lázár, Gábor Jancsó, Laura Pálvölgyi, Ildikó Dobos, István Nagy, Péter Sántha

**Affiliations:** ^1^Department of Psychiatry, Faculty of Medicine, University of Szeged, Szeged, Hungary; ^2^Department of Physiology, University of Szeged, Szeged, Hungary; ^3^Department of Surgery and Cancer, Faculty of Medicine, Imperial College London, London, United Kingdom

**Keywords:** insulin, neurite outgrowth, dorsal root ganglion neurons, insulin receptor, transient receptor potential vanilloid type 1 receptor, calcitonin gene-related peptide, isolectin B4

## Abstract

Apart from its pivotal role in the regulation of carbohydrate metabolism, insulin exerts important neurotrophic and neuromodulator effects on dorsal root ganglion (DRG) neurons. The neurite outgrowth-promoting effect is one of the salient features of insulin’s action on cultured DRG neurons. Although it has been established that a significant population of DRG neurons express the insulin receptor (InsR), the significance of InsR expression and the chemical phenotype of DRG neurons in relation to the neurite outgrowth-promoting effect of insulin has not been studied. Therefore, in this study by using immunohistochemical and quantitative stereological methods we evaluated the effect of insulin on neurite outgrowth of DRG neurons of different chemical phenotypes which express or lack the InsR. Insulin, at a concentration of 10 nM, significantly increased total neurite length, the length of the longest neurite and the number of branch points of cultured DRG neurons as compared to neurons cultured in control medium or in the presence of 1 μM insulin. In both the control and the insulin exposed cultures, ∼43% of neurons displayed InsR-immunoreactivity. The proportions of transient receptor potential vanilloid type 1 receptor (TRPV1)-immunoreactive (IR), calcitonin gene-related peptide (CGRP)-IR and *Bandeiraea simplicifolia* isolectin B4 (IB4)-binding neurons amounted to ∼61%, ∼57%, and ∼31% of DRG neurons IR for the InsR. Of the IB4-positive population only neurons expressing the InsR were responsive to insulin. In contrast, TRPV1-IR nociceptive and CGRP-IR peptidergic neurons showed increased tendency for neurite outgrowth which was further enhanced by insulin. However, the responsiveness of DRG neurons expressing the InsR was superior to populations of DRG neurons which lack this receptor. The findings also revealed that besides the expression of the InsR, inherent properties of peptidergic, but not non-peptidergic nociceptive neurons may also significantly contribute to the mechanisms of neurite outgrowth of DRG neurons. These observations suggest distinct regenerative propensity for differing populations of DRG neurons which is significantly affected through insulin receptor signaling.

## Introduction

Insulin, besides being essential in the regulation of glucose homeostasis, is involved in several neuronal processes such as neural survival and neurite outgrowth in both the central and the peripheral nervous system (Fernyhough et al., 1993; Wan et al., 1997; Barber et al., 2001; Stella et al., 2001; Gerozissis, 2003; Singh et al., 2012). Insulin exerts its neural actions through the insulin receptor (InsR), a member of the receptor tyrosine kinases family (Ward and Lawrence, 2009) present in nerve tissue (Havrankova et al., 1978; Unger et al., 1989). Further, it has also been revealed that a relatively high proportion of dorsal root ganglion (DRG) neurons express the InsR (Baiou et al., 2007; Lázár et al., 2018a,b).

Previous studies demonstrated that a unique population of C-fiber DRG neurons is sensitive to capsaicin and express the transient receptor potential vanilloid type 1 receptor (TRPV1) (Jancsó et al., 1977; Buck and Burks, 1986; Holzer, 1991; Caterina et al., 1997; Guo et al., 2001). These neurons are involved in pain sensation and, in a variety of organs, local regulatory functions, including neurogenic inflammation brought about by the release of sensory neuropeptides such as substance P (SP) and calcitonin gene-related peptide (CGRP) (Jancsó, 1960; Jancso et al., 1968, Jancsó et al., 1977, 2009; Maggi and Meli, 1988; Holzer, 1991; Nagy et al., 2004). These TRPV1-immunoreactive (IR) nociceptive DRG neurons comprise two major subpopulations: CGRP-containing peptidergic and *Bandeiraea simplicifolia* isolectin B4 (IB4)-binding non-peptidergic neurons (Silos-Santiago et al., 1995; Santha et al., 2010). It has also been shown that peptidergic and non-peptidergic sensory neurons have distinct sensitivities to neurotrophic factors such as nerve growth factor (NGF) and glial cell derived neurotrophic factor (GDNF) (Molliver et al., 1997).

Recent findings indicated that insulin may modulate the activation of nociceptive sensory neurons through indirectly opening the TRPV1 (Sathianathan et al., 2003; Van Buren et al., 2005; Lilja et al., 2007). Immunohistochemical studies have also revealed that a high proportion of InsR-IR DRG neurons show colocalization with TRPV1 and CGRP in rat and mouse DRG neurons (Baiou et al., 2007; Lázár et al., 2018a,b). It has also been suggested that an interplay among insulin, InsR and TRPV1 in nociceptive DRG neurons may contribute to the mechanisms of inflammatory processes (Razavi et al., 2006; Tsui et al., 2007; Lázár et al., 2018a,b).

Additionally, InsR signaling has been shown to affect neurite outgrowth in cultured sympathetic and sensory neurons, but the nature of change, an increase or decrease in neurite outgrowth is concentration dependent (Recio-Pinto et al., 1986; Fernyhough et al., 1993; Singh et al., 2012). Insulin signaling in sensory neurons has also been suggested to participate in pathophysiological alterations induced by diabetes. Available experimental evidence indicates that insulin not only promotes peripheral nerve regeneration *in vivo* (Xu et al., 2004; Toth et al., 2006), but may prevent diabetes-induced functional deficits, too (Singhal et al., 1997). Although these studies furnished evidence for a pro-regenerative effect of insulin on sensory neurons, a possible association of the neuronal phenotype with the neurite outgrowth-promoting effect of insulin has not been evaluated.

Therefore, the present study was initiated in an attempt to characterize the phenotypic traits of DRG neurons responsive to insulin’s neurite outgrowth-promoting action. To achieve this goal, selected parameters of neurite outgrowth of cultured DRG neurons which expressed the InsR, TRPV1, CGRP and/or bound the IB4 were assessed in the presence or absence of insulin with immunohistochemical and quantitative stereological methods.

## Materials and Methods

All experiments were approved by the Ethics Committee for Animal Care of the University of Szeged and were carried out in full accordance with the Directive 2010/63/EU of the European Parliament and of the Council on the Protection of Animals Used for Scientific Purposes and the guidelines of the Committee for Research. All efforts were made to minimize animal suffering. The number of experimental animals was kept as low as possible.

### Preparation of Cultures of DRG Neurons

Adult male Wistar rats weighing 300–350 g (*n* = 12) were terminally anesthetized and DRGs were removed under aseptic conditions. DRGs were collected in a culture medium (Dulbecco’s Modified Eagle’s Medium/Nutrient mixture F-12 HAM; Sigma-Aldrich, Gillingham, United Kingdom) containing 1 mM l-glutamine, 50 IU/ml penicillin, 50 μg/ml streptomycin, and 300 mg/dL glucose (all from Sigma-Aldrich) and 4% Ultroser G (BioSepra SA, Cergy, France) without insulin. DRG neuron cultures were prepared as described previously (Winter et al., 1988; Santha et al., 2010). Briefly, DRGs were incubated with collagenase type IV (2000 U/ml, Sigma-Aldrich) for 3 h at 37°C in a 5% CO_2_ incubator. DRGs were triturated with a fire-polished Pasteur pipette. The cell suspension was spun through 21% Percoll^®^ (Sigma-Aldrich). Following re-suspension in the supplemented culture medium, DRG cells were plated on poly-DL-ornithine (Sigma-Aldrich) coated glass coverslips and kept at 37°C in a 5% CO_2_ atmosphere. Insulin (Sigma-Aldrich) was administered to the culture medium at concentrations of 10 nM and 1 μM for 48 h. The concentrations of insulin applied in the present experiments are similar to those found in commonly used serum-free media (cf. Chen et al., 2008), importantly, these concentrations of insulin were applied in previous studies as well (Recio-Pinto et al., 1986; Fernyhough et al., 1993; Singh et al., 2012). To determine the survival ratios of DRG neurons among each experimental group, the ethidium homodimer-1 assay for cell viability (LIVE/DEAD^TM^ Viability Kit; Invitrogen, Carlsbad, CA, United States) was performed.

### Immunohistochemistry

DRG cultures were fixed with 4% paraformaldehyde in 0.1 M phosphate buffer (pH 7.4) for 10 min and then washed twice in phosphate-buffered isotonic NaCl solution (phosphate-buffered saline, PBS). In single and multiple labeling experiments, the following primary antibodies and lectins were used: mouse anti-β3-tubulin (Santa Cruz Biotechnologies, Dallas, TX, United States; 1:1500), rabbit polyclonal anti-InsRα subunit IgG (Santa Cruz Biotechnologies, 1:1500), polyclonal guinea pig anti-TRPV1 IgG (Neuromics, Edina, MN, United States; 1:1500), polyclonal goat anti-CGRP IgG (Santa Cruz Biotechnologies; 1:5000) and biotin-conjugated IB4 (Sigma-Aldrich, 1:1000). Primary antibodies were dissolved in PBS containing 0.3% Triton X-100 and applied to the cultures overnight at 4°C. After rinsing in PBS, the following secondary antibodies were applied: donkey anti-rabbit IgG labeled with DL488 (1:500), donkey anti-guinea pig IgG labeled with Cy3 (1:500) and AMCA-avidin conjugates were used as secondary antibodies (all from Jackson Immunoresearch Laboratories, West Grove, PA, United States). After incubation for 2 h, the specimens were rinsed in PBS and covered with Prolong Gold antifade mounting medium (Invitrogen). The selectivity and specificity of the polyclonal rabbit anti-TRPV1 and anti-InsR antisera (Santa Cruz Biotechnologies) were assessed in DRG sections by the lack of staining with the TRPV1 antibody in specimens obtained from TRPV1^-/-^ mice and by the failure of staining with the InsR antibody after preincubation with the immunizing peptide supplied by the manufacturer (Baiou et al., 2007). Control procedures for CGRP immunolabeling was performed by replacing the primary antiserum with normal donkey serum. No immunostaining was observed in control experiments.

### Quantitative Assessment of Neurite Outgrowth of Cultured DRG Neurons

Photomicrographs were taken with a Leica DMLB fluorescence microscope (Wetzlar, Germany) equipped with a Retiga 2000R digital camera (QImaging, Surrx, BC, Canada). In control and insulin-treated cultures, at least 30 neurons per culture were counted and analyzed. Data collection was performed blinded to the experimental conditions. Each image was analyzed using ImagePro Plus 7 image processing and analysis software (Media Cybernetics, Bethesda, MD, United States) and ImageJ image analysis software (NIH, Bethesda, MD, United States). Neurons exhibiting IB4 binding and immunoreactivity for the InsR, TRPV1, and CGRP or a combination of these molecular markers were counted. To assess the effect of insulin on neurite outgrowth, first, we examined the percentages of neurite-bearing DRG neurons in the control and the insulin-treated cultures. Thereafter, by using the ImageJ (NIH) simple neurite tracer program, we measured three quantified parameters of neurons with neurites longer than the soma diameter and clearly identifiable axonal arborization: total neurite length (sum of lengths of all neurites), maximum neurite length (length of longest neurite) and neurite branch points (number of neurite branch points).

### Statistical Analysis

Data were collected from three to nine independent experiments. All data are presented as the mean ± standard error of the mean (SEM). To test the normality of the distribution of data in each experimental group, the Shapiro–Wilk test was applied. Statistical comparisons of data were performed with Fisher’s exact probability test or a one-way ANOVA with *post hoc* Tukey’s multiple comparison test, as appropriate. A *p*-value of less than 0.05 was considered to be statistically significant for all tests. Statistical analysis was made by using the Dell Statistica software (Dell Inc., Tulsa, OK, United States).

## Results

### Insulin Increases Neurite Outgrowth of Cultured Adult Rat DRG Neurons at Nanomolar Concentration

The morphology of cultured DRG neurons was studied in specimens stained with the mouse anti-β3-tubulin antibody. The survival ratios of DRG neurons were not significantly altered by insulin and the high glucose concentration of the medium as assessed with the ethidium homodimer-1 assay. The percentages of dead cells amounted to 3.01 ± 0.34%, 2.47 ± 0.21%, and 4.13 ± 0.18% in the cultures exposed to culture medium and insulin at a concentrations of 10 nM and 1 μM. The differences between each experimental groups were not significant (*p* < 0.05). These observations are in accord with previous findings showing the lack of a significant effect of insulin on neuronal counts in DRG cultures (Fernyhough et al., 1993). Similarly, the high concentration of glucose in the DMEM/F12 medium has been shown to induce oxidative stress and neuronal degeneration in cultured DRG neurons of streptozotocin diabetic but not control rats (Zherebitskaya et al., 2009).

**FIGURE 1:**
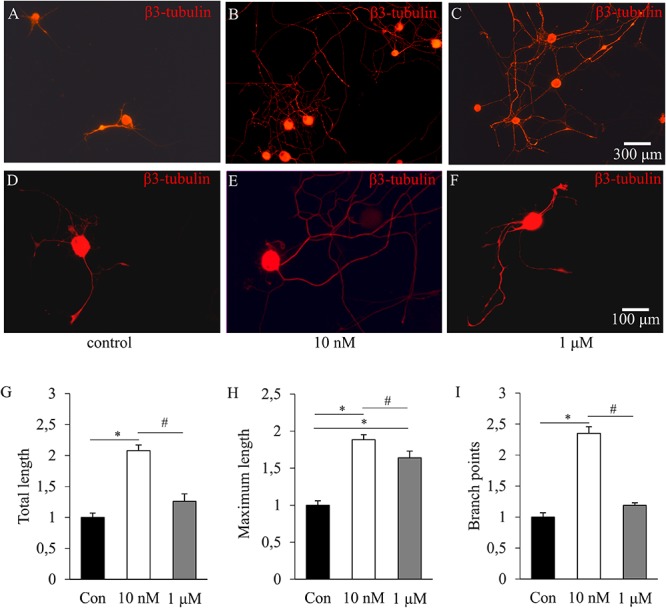
The effects of insulin on neurite outgrowth of cultured adult rat dorsal root ganglion (DRG) neurons. **(A–F)** Fluorescence photomicrographs of β3-tubulin-stained DRG neurons illustrating the effects of insulin applied at concentrations of 10 nM **(B)** and 1 μM **(C)**. The scale bar in **(C)** indicates 300 μm and applies to photomicrographs **(A–C)**, the scale bar in **(F)** indicates 100 μm and applies to photomicrographs **(D–F)**. **(G–I)** Quantitative morphometric evaluation of the effects of insulin on neurite outgrowth. Changes in total neurite length **(G)**, maximum neurite length **(H)**, and number of branch points **(I)** are shown as fold increase of the control. Values are expressed as mean ± standard error of the mean (SEM). ^∗^Statistically significantly different from the control (*p* < 0.05). ^#^Parameters of DRG neurons treated with 10 nM and 1 μM insulin, respectively, are significantly different (*p* < 0.05).

Administration of insulin at concentrations of 10 nM and 1 μM significantly increased the percentages of neurite-bearing DRG neurons by 50.74 ± 2.52% and 26.86 ± 1.78%, respectively, as compared to controls (*p* < 0.05). The difference between the cultures exposed to 10 nM and 1 μM were also significant (*p* < 0.05). Neurite outgrowth was examined by analyzing three quantified parameters of neurons with neurites longer than the soma diameter and clearly identifiable axonal arborization: total neurite length (sum of lengths of all neurites), maximum neurite length (length of the longest neurite), and neurite branch points (number of neurite branch points). The results showed that administration of insulin at a concentration of 10 nM for 48 h increased total neurite length by 2.08-fold, maximum neurite length by 1.88-fold and neurite branch points by 2.35-fold as compared to control cultures (for all comparisons *p* < 0.05; **Figures 1A–I**). Administration of insulin, at a concentration of 1 μM, increased maximum neurite length by 1.64-fold (*p* < 0.05), whereas no significant changes were observed in total neurite length and neurite branch points (**Figures 1A–I**). Therefore, only the effects of insulin applied at a concentration of 10 nM were analyzed in detail in this study.

### Distinct Neurite Growth-Promoting Effect of Insulin on Neurochemically Differing Subpopulations of DRG Neurons

**FIGURE 2:**
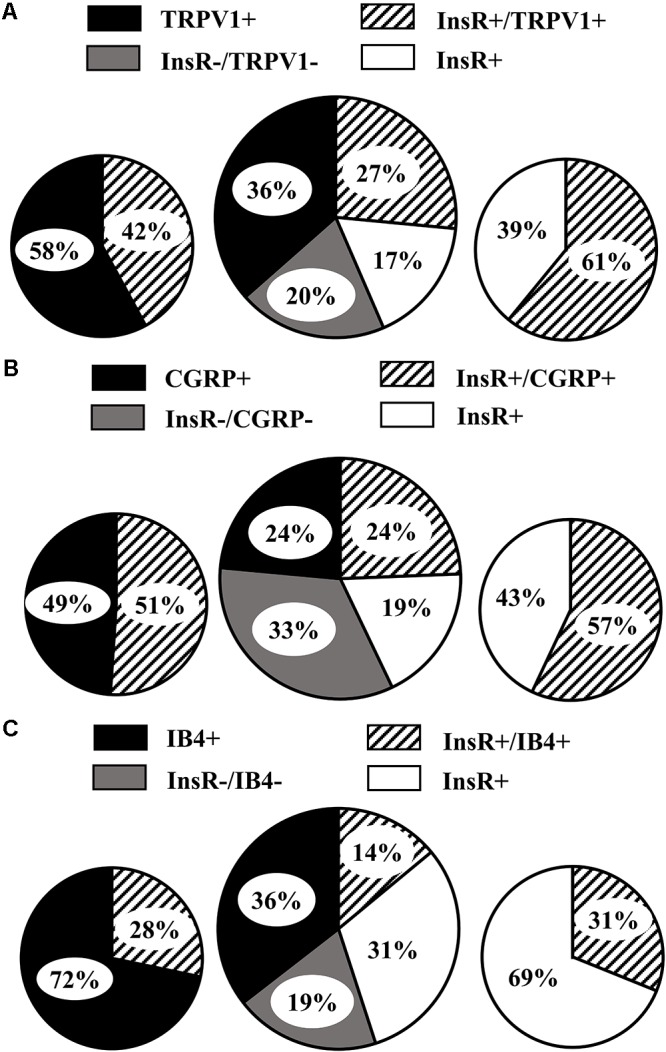
Neurochemical phenotypes of cultured adult rat dorsal root ganglion (DRG) neurons examined. **(A)** Large pie chart shows the percentage distribution of populations of DRG neurons showing immunoreactivities for the insulin receptor (InsR) and the transient receptor potential vanilloid type 1 receptor (TRPV1). Small pie charts show the relative proportions of TRPV1-immunoreactive (IR) neurons which display colocalization with the InsR and, conversely, the relative proportions of InsR-IR DRG neurons which display colocalization with TRPV1. **(B)** Large pie chart shows the percentage distribution of populations of DRG neurons showing immunoreactivities for the InsR and calcitonin gene-related peptide (CGRP). Small pie charts show the relative proportions of CGRP-IR DRG neurons which display colocalization with the InsR and, conversely, the relative proportions of InsR-IR DRG neurons which display colocalization with CGRP. **(C)** Large pie chart shows the percentage distribution of populations of DRG neurons showing immunoreactivity for the InsR and binding of the isolectin B4 (IB4). Small pie charts show the relative proportions of IB4-binding neurons which display colocalization with the InsR and, conversely, the relative proportions of InsR-IR DRG neurons which display colocalization with IB4.

**FIGURE 3:**
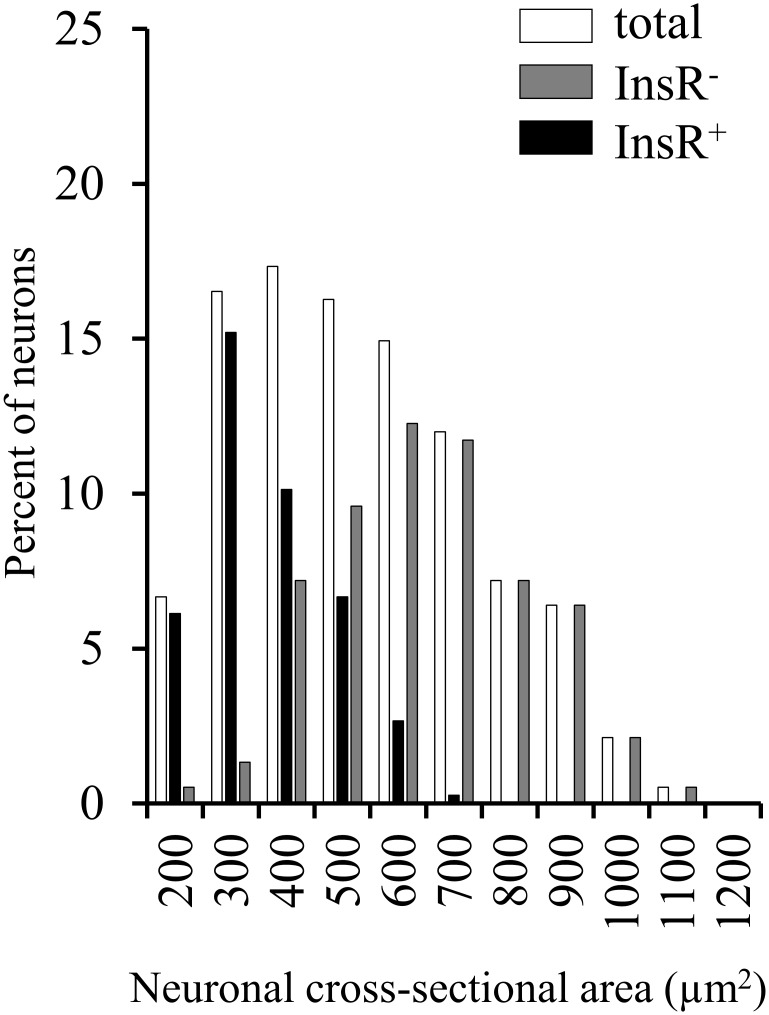
Size-frequency distribution histogram of the total and the insulin receptor (InsR)-immunopositive and -negative populations of adult rat cultured dorsal root ganglion (DRG) neurons. Size-frequency distribution histogram shows the cross-sectional areas of the total DRG neuronal population (white bars), the InsR-immunopositive (black bars) and the InsR-immunonegative (gray bars) populations in the control cultures.

Neurochemically distinct subpopulations of cultured DRG neurons were assessed by counting DRG neurons which showed immunoreactivity for the InsR, TRPV1, CGRP, or IB4 binding, respectively. In control cultures, 43.68 ± 1.71% of DRG neurons were IR for the InsR (**Figure 2**). The size-frequency distribution of the InsR-IR neurons revealed that InsR-positive DRG neurons are small-medium sized neurons. The mean cross-sectional area of the InsR-IR and InsR-immunonegative DRG neurons amounted to 321.11 ± 11.2 μm^2^ and 526.39 ± 15.22 μm^2^, respectively (**Figure 3**). Of the TRPV1-, CGRP-, and IB4-positive DRG neurons 63.09 ± 2.42%, 48.13 ± 2.37%, and 49.58 ± 3.7%, respectively, showed InsR-IR (**Figure 2**). Administration of insulin at concentrations of 10 nM and 1 μM did not significantly alter the percentage distribution of InsR-, TRPV1-, and CGRP-IR, and IB4-binding DRG neurons (*p* < 0.05). Analysis of the TRPV1- and the CGRP-IR and the IB4-binding populations revealed that 60.92 ± 3.38%, 56.98 ± 2.81%, and 31.11 ± 3.7% of the InsR-IR neurons were TRPV1-, CGRP-, and IB4-positive, respectively (**Figure 2**). The proportions of TRPV1-IR, CGRP-IR, and IB4-binding neurons expressing the InsR were significantly different (*p* < 0.05). The InsR was expressed in 42.06 ± 3.39%, 51.04 ± 4.26%, and 28.29 ± 2.4% of the TRPV1-IR, CGRP-IR, and IB4-binding neurons, respectively (**Figure 2**). The percentages of neurons which colocalized the InsR with TRPV1, CGRP, and IB4 amounted to 26.5 ± 2.17%, 24.33 ± 3.25%, and 13.82 ± 2.37%, respectively (**Figure 2**).

To reveal the neurite outgrowth-promoting effect of insulin on different subpopulations of DRG neurons, the three quantified parameters of InsR-, TRPV1-, and CGRP-IR and IB4-binding DRG neurons were analyzed in control and insulin-treated cultures. Photomicrographs in **Figures 5A–C, 6A–C, 7A–C** illustrate examples of analyzed InsR and TRPV1-IR, InsR- and CGRP-IR, and InsR-IR and IB4-binding populations of cultured DRG neurons exposed to insulin (10 nM).

**FIGURE 4:**
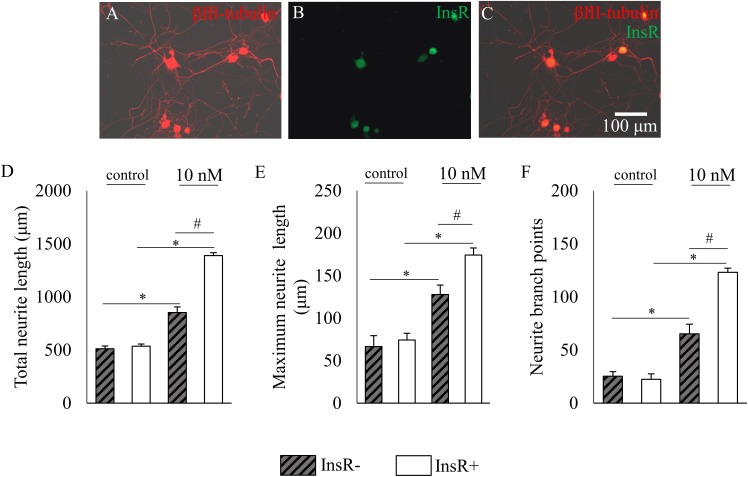
The effects of insulin (10 nM, 48 h) on neurite outgrowth of insulin receptor (InsR)-positive and InsR-negative cultured adult rat dorsal root ganglion (DRG) neurons. **(A–C)** Fluorescence photomicrographs of cultured adult rat dorsal root ganglion (DRG) neurons double-stained for β3-tubulin- and InsR-immunoreactivities. The scale bar in **(C)** indicates 100 μm and applies to all photomicrographs. **(D–F)** Quantitative morphometric evaluation of the effects of insulin on neurite outgrowth of InsR-positive and InsR-negative populations of DRG neurons. Changes in total neurite length **(D)**, maximum neurite length **(E)** and number of branch points **(F)** are shown. Values are expressed as mean ± standard error of the mean (SEM). ^∗^Statistically significantly different from the control (*p* < 0.05). ^#^Parameters of InsR-positive and InsR-negative DRG neurons are significantly different (*p* < 0.05).

Our data revealed that insulin significantly increased the total neurite length from 513.21 ± 25.86 μm to 853.95 ± 52.65 μm, the maximum neurite length from 66.57 ± 12.9 μm to 127.88 ± 11.21 μm, and the neurite branch points from 25.38 ± 4.32 to 65.38 ± 9.02 in the InsR-immunonegative neuronal population (*p* < 0.05). Our data also demonstrated that in InsR-IR neurons, insulin significantly increased the total neurite length from 536.61 ± 20.29 μm to 1390.21 ± 27.17 μm, the maximum neurite length from 74.42 ± 7.74 μm to 174.33 ± 8.36 μm, and the neurite branch points from 22.56 ± 5.14 to 123.15 ± 4.0, respectively (for all comparisons *p* < 0.05; **Figures 4A–F**). Statistical analysis revealed that differences between each quantified parameter of insulin-treated neuronal populations which express or lack the InsR were significant (*p* < 0.05).

**FIGURE 5:**
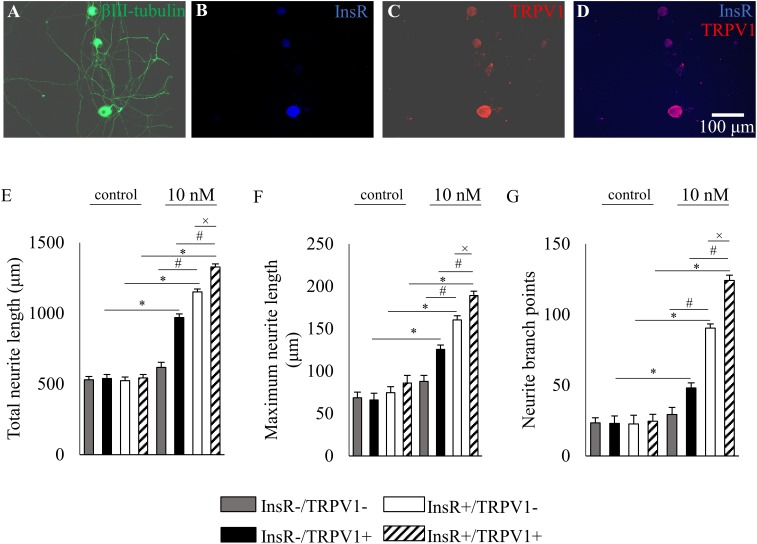
The effects of insulin (10 nM, 48 h) on neurite outgrowth of cultured adult rat dorsal root ganglion (DRG) neurons. **(A–D)** Fluorescence photomicrographs of cultured adult rat dorsal root ganglion (DRG) neurons triple-stained for β3-tubulin, insulin receptor (InsR) and transient receptor potential vanilloid type 1 receptor (TRPV1). The scale bar in **(D)** indicates 100 μm and applies to all photomicrographs. **(E–G)** Quantitative morphometric evaluation of the effects of insulin on neurite outgrowth of InsR-positive and InsR-negative populations of TRPV1-positive and TRPV1-negative DRG neurons. Changes in total neurite length **(E)**, maximum neurite length **(F)**, and number of branch points **(G)** are shown. Values are expressed as mean ± standard error of the mean (SEM). ^∗^, ^#^, and ^x^: Statistically significantly different from the corresponding control and/or insulin-treated DRG neuron populations (*p* < 0.05).

Our morphological analysis also revealed that insulin significantly increased total neurite length from 539.02 ± 27.91 μm to 969.45 ± 25.62 μm, the maximum neurite length from 65.85 ± 8.01 μm to 125.59 ± 5.04 μm, and the neurite branch points from 22.91 ± 5.32 to 48.04 ± 3.65 of neurons which were IR for TRPV1, but not for the InsR (*p* < 0.05; **Figures 5A–G**). Conversely, in neurons which displayed the InsR, but not the TRPV1, insulin significantly increased the total neurite length from 522.98 ± 26.44 μm to 1150.89 ± 21.01 μm, the maximum neurite length from 74.426 ± 7.22 μm to 160.36 ± 4.77 μm, and the neurite branch points from 22.56 ± 6.2 to 90.38 ± 2.99, respectively (*p* < 0.05; **Figures 5A–G**). Statistical analysis revealed that differences between each quantified parameter of insulin-treated neuronal populations which express or lack the InsR were significant (*p* < 0.05). In DRG neurons which showed colocalization of the InsR and TRPV1, insulin significantly increased the total neurite length from 542.08 ± 25.17 μm to 1326.97 ± 22.77 μm, the maximum neurite length from 85.84 ± 9.02 μm to 188.93 ± 5.2 μm, and the neurite branch points from 24.56 ± 4.88 to 124.05 ± 3.84, respectively (for all comparisons *p* < 0.05; **Figures 5A–G**). Statistical analysis revealed that differences between each quantified parameter of insulin-treated InsR-IR neuronal populations which express or lack the TRPV1 were significant (*p* < 0.05).

**FIGURE 6:**
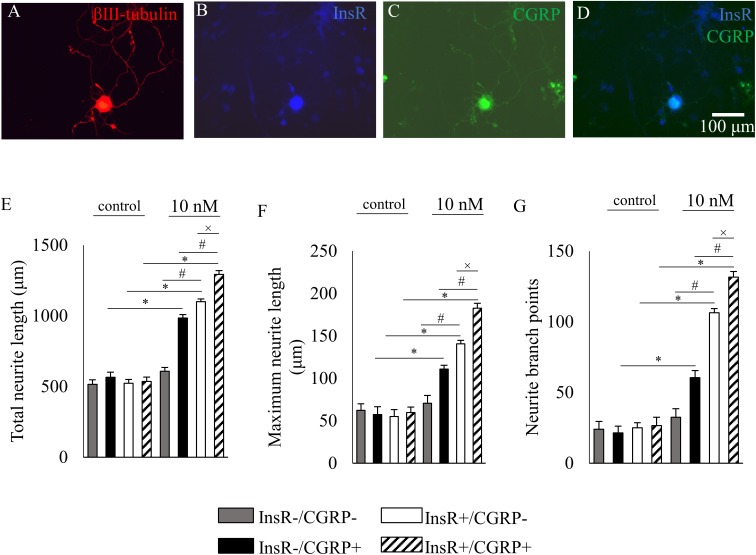
The effects of insulin (10 nM, 48 h) on neurite outgrowth of cultured adult rat dorsal root ganglion (DRG) neurons. **(A–D)** Fluorescence photomicrographs of cultured adult rat dorsal root ganglion (DRG) neurons triple-stained for β3-tubulin, insulin receptor (InsR) and calcitonin gene-related peptide (CGRP). The scale bar in **(D)** indicates 100 μm and applies to all photomicrographs. **(E–G)** Quantitative morphometric evaluation of the effects of insulin on neurite outgrowth of InsR-positive and InsR-negative populations of CGRP-positive and CGRP-negative DRG neurons. Changes in total neurite length **(E)**, maximum neurite length **(F)** and number of branch points **(G)** are shown. Values are expressed as mean ± standard error of the mean (SEM). ^∗^, ^#^, and ^x^: Statistically significantly different from the corresponding control and/or insulin-treated DRG neuron populations (*p* < 0.05).

Our data also showed that in the CGRP-IR and InsR-immunonegative DRG neuronal population, insulin significantly increased the total neurite length from 565.47 ± 3.94 μm to 984.33 ± 24.03 μm, the maximum neurite length from 57.4 ± 9.06 μm to 110.81 ± 4.54 μm, and the neurite branch points from 21.47 ± 4.73 to 60.5 ± 4.99, respectively (for all comparisons *p* < 0.05; **Figures 6A–G**). In the InsR-IR and CGRP-immunonegative DRG neurons, insulin significantly increased the total neurite length from 522.99 ± 26.59 μm to 1099.66 ± 19.77 μm, the maximum neurite length from 54.99 ± 8.01 μm to 140.67 ± 3.99 μm, and the neurite branch points from 24.97 ± 3.66 to 106.37 ± 3.01, respectively (for all comparisons *p* < 0.05; **Figures 6A–G**). Statistical analysis revealed that differences between each quantified parameter of insulin-treated neuronal populations which express or lack the InsR were significant (*p* < 0.05). In InsR- and CGRP-IR neurons insulin significantly increased all the three quantified parameters: the total neurite length from 535.62 ± 30.6 μm to 1292.86 ± 26.88 μm, the maximum neurite length from 59.67 ± 6.44 μm to 182.55 ± 5.8 μm, and the neurite branch points from 26.55 ± 5.89 to 131.55 ± 3.99 (for all comparisons *p* < 0.05; **Figures 6E–G**). Statistical analysis revealed that differences between each quantified parameter of insulin-treated InsR-IR neuronal populations which express, or lack CGRP-IR were significant (*p* < 0.05). The statistical analysis revealed that the differences between the treated populations were also significant (*p* < 0.05).

**FIGURE 7:**
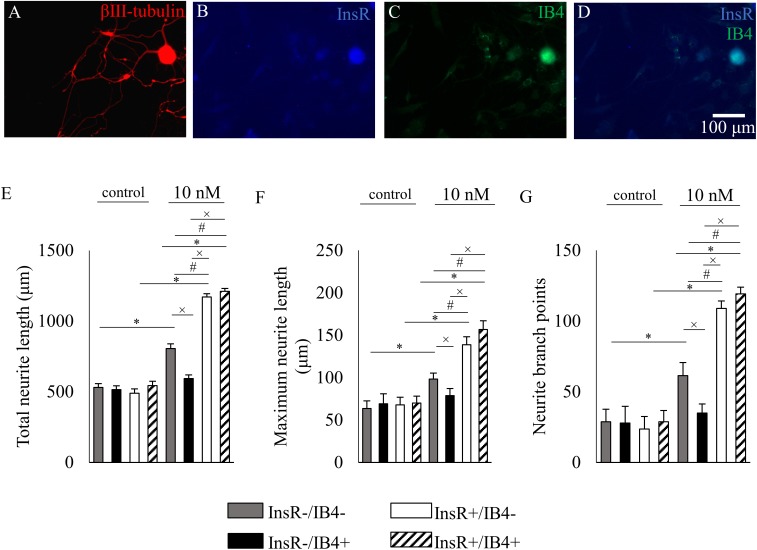
The effects of insulin (10 nM, 48 h) on neurite outgrowth of cultured adult rat dorsal root ganglion (DRG) neurons. **(A–D)** Fluorescence photomicrographs of cultured adult rat dorsal root ganglion (DRG) neurons triple-stained for β3-tubulin, insulin receptor (InsR) and isolectin B4 (IB4). The scale bar in **(D)** indicates 100 μm and applies to all photomicrographs. **(E–G)** Quantitative morphometric evaluation of the effects of insulin on neurite outgrowth of InsR-positive and InsR-negative populations of IB4-positive and IB4-negative DRG neurons. Changes in total neurite length **(E)**, maximum neurite length **(F)** and number of branch points **(G)** are shown. Values are expressed as mean ± standard error of the mean (SEM). ^∗^, ^#^, and ^x^: Statistically significantly different from the corresponding control and/or insulin-treated DRG neuron populations (*p* < 0.05).

Analysis of the insulin responsiveness of the IB4-positive DRG neurons revealed that neurons which lack the InsR failed to show changes in any of the quantified parameters examined (**Figures 7A–G**). In contrast, in IB4-positive neurons displaying the InsR, insulin treatment significantly increased total neurite length from 543.56 ± 30.96 μm to 1210.81 ± 20.08 μm, the maximum neurite length from 70.3 ± 7.99 μm to 156.81 ± 10.1 μm, and neurite branch points from 28.67 ± 7.99 to 119.21 ± 4.7, respectively (for all comparisons *p* < 0.05; **Figures 7A–G**).

## Discussion

Insulin exerts neurotrophic and neuromodulatory effects in a variety of central and peripheral neuronal systems (Recio-Pinto et al., 1986; Fernyhough et al., 1993; Wan et al., 1997; Barber et al., 2001; Stella et al., 2001; Gerozissis, 2003; Sathianathan et al., 2003; Singh et al., 2012). The present data support and extend previous observations by showing that nanomolar concentrations of insulin significantly increase neurite outgrowth of cultured adult rat DRG neurons (Recio-Pinto et al., 1986; Fernyhough et al., 1993; Singh et al., 2012). In accord with earlier studies, the present findings also demonstrate the disparate effects of low (nM) and high (μm) concentrations of insulin on neurite outgrowth (Recio-Pinto et al., 1986; Fernyhough et al., 1993; Singh et al., 2012). However, the most important finding of the present study is that neurochemically distinct populations of DRG neurons display differing responsiveness to the neurite outgrowth-promoting effect of insulin.

Adult rat DRG neurons can be classified on the basis of their sensitivity to and dependence on neurotrophins. During the postnatal period both peptidergic and non-peptidergic DRG neurons are responsive to NGF and express the tropomyosin receptor kinase A (TrkA). However, in the course of their development, expression of TrkA is downregulated and non-peptidergic, IB4-binding neurons lose their NGF-sensitivity and become sensitive to GDNF (Molliver et al., 1997). Several studies demonstrated significant changes in the expression of proteins and peptides in DRG neurons following axonal injuries or culturing (cf. Jancso, 1992; Hokfelt et al., 1994). However, in the present study morphometric analysis of CGRP- and IB4-positive neurons in control and insulin-treated cultures revealed percentage distributions similar to control ganglia *in vivo* (e.g., Lindsay et al., 1989; Hjerling-Leffler et al., 2007). This is in agreement with previous findings showing that the time frame of changes in the expression pattern of DRG neurons which commence in response to injury or exposure to trophic factors is longer than that in the present experiments (cf. Lindsay et al., 1989; Hokfelt et al., 1994).

The expression of the InsR has been demonstrated in a relatively high proportion of DRG neurons in both rats and mice (Baiou et al., 2007; Lázár et al., 2018a,b). The present findings confirm these observations by showing that up to 45% of cultured adult rat DRG neurons, which comprise both peptidergic and IB4-binding DRG neurons, display InsR-immunoreactivity. Further, most TRPV1-expressing neurons, which involve both peptidergic and non-peptidergic populations, also express the InsR. The present results are in agreement with previous *in vitro* and *in vivo* findings by showing that InsR-IR DRG neurons are small-medium sized (Baiou et al., 2007; Lázár et al., 2018a,b).

The results demonstrate that populations of DRG neurons which express the InsR show an increased propensity for neurite outgrowth as compared to populations which lack this receptor. This is especially evident in the IB4-binding population of neurons; only those neurons which express the InsR exhibit an increase in neurite outgrowth upon insulin exposure. The exact mechanism of insulin-induced increased neurite outgrowth is not clearly established, but the role of the InsR has been implicated (Singh et al., 2012; Grote et al., 2013; Grote and Wright, 2016). It has been proposed that common signaling mechanisms may contribute to the activation of both the InsR and some classic neurotrophins such as NGF (Brewster et al., 1994; Grote et al., 2013; Grote and Wright, 2016). Possible molecular mechanisms of insulin’s neurite outgrowth promoting action involve stabilization of tubulin, an essential component of axon and dendrite growth (Fernyhough et al., 1989). The role of insulin at low (nM) concentrations in the activation of the phosphoinositide 3-kinase/protein kinase B (PI3K-Akt) signaling pathway (Huang et al., 2005), which is critically involved in the mechanisms of axonal growth (Goldberg, 2003), has also been revealed. Previous observations have also shown that high (μM) concentrations of insulin, through the desensitization of the InsRs, render DRG neurons less sensitive to the neurotrophic effects of insulin by the inhibition of the PI3K-Akt pathway (Singh et al., 2012). In addition, increase in GSK-3β levels may also significantly contribute to the decreased sensitivity and, consequently impaired growth cone advancement (Eickholt et al., 2002; Singh et al., 2012).

However, other specific traits, such as the expression of TRPV1 and/or CGRP also significantly modulate neurite outgrowth. Hence, although the results suggest that the expression of the InsR enhances neurite outgrowth in all populations of DRG neurons examined, the expression of TRPV1 and/or CGRP *per se* appear to promote neurite outgrowth. Signaling mechanisms involved in the mediation of the neurite outgrowth-promoting effect of insulin may share common pathways contributing to the expression and sensitization of the TRPV1 by NGF (Winter et al., 1988; Brewster et al., 1994; Shu and Mendell, 1999; Nicol and Vasko, 2007; Grote et al., 2013; Grote and Wright, 2016). It has been demonstrated that the NGF receptor, TrkA plays an important role in insulin signaling in PC12 cells which share characteristics with sensory neurons (Geetha et al., 2013). Hence, it has been shown that insulin can induce the phosphorylation of TrkA which is essential in the activation of the InsR in PC12 cells (Geetha et al., 2013). CGRP-IR neurons are also sensitive to and dependent on NGF: it plays a critical role in the survival of CGRP-IR neurons in early development and regulates peptide expression and neurite outgrowth in the adult (Lindsay et al., 1989; Molliver et al., 1997). It can be hypothesized that in CGRP-IR neurons which lack the InsR, insulin promotes neurite outgrowth through TrkA signaling, instead of insulin signaling via the InsR. Further, CGRP-IR DRG neurons have been shown to be increasingly sensitive to high concentrations of both insulin and NGF under pathological conditions (Takatori et al., 2008; Zamami et al., 2011; Hashikawa-Hobara et al., 2012). Taken together, these findings may suggest that NGF sensitivity of the TRPV1- and CGRP-IR DRG neurons could explain their inherent propensity for increased neurite outgrowth. This assumption is supported by the apparent insensitivity to insulin’s neurite outgrowth promoting effect of the IB4-positive neurons which lack both the InsR and sensitivity to NGF. Furthermore, novel findings in sensory neuron InsR knockout mice suggested that not the InsR itself, but an interaction between insulin, TRPV1 and/or neuropeptides, such as CGRP is crucial in the development of dysfunctions of insulin signaling in DRG neurons (Grote et al., 2018). However, the results demonstrate a significantly higher expression rate of the InsR in TRPV1- and CGRP-IR DRG neurons as compared to the IB4-positive population. Importantly, the responsiveness of DRG neurons expressing the InsR was superior to populations of DRG neurons which lack this receptor, suggesting a pivotal role of InsR signaling in the mechanisms of neurite outgrowth.

Importantly, it has also been reported that similar to NGF application, insulin treatment facilitates the formation of collateral sprouting of small diameter neurons (Diamond et al., 1992; Singhal et al., 1997; Guo et al., 2011). The present findings are in accord with this observation and, in addition, demonstrate that branch points were particularly increased by insulin in InsR-IR neurons. These observations may suggest that InsR signaling may bear of importance in the mechanism of collateral sprouting of sensory neurons as well.

## Conclusion

The present experiments confirm and extend previous findings on the neurite outgrowth-promoting effect of insulin and the expression of the InsR in cultured adult rat DRG neurons. The most important new observation of this study is that neurons which express the InsR exhibit a significantly higher propensity for the neurite outgrowth-promoting effect of insulin as compared to neurons which lack this particular receptor. Further, the data indicate that peptidergic CGRP-IR and TRPV1-IR nociceptive neurons expressing the InsR may be identified as the major subpopulations of DRG neurons which exhibit insulin-induced increased neurite outgrowth. The significance of the InsR in promoting neurite outgrowth is further supported by the observation that insulin increased neurite outgrowth only in a subpopulation of IB4-positive neurons which express the InsR. These findings suggest distinct regenerative propensity of differing populations of DRG neurons which is significantly affected through InsR signaling. In addition, the different responsiveness of separate chemotypes of primary sensory neurons to insulin may interfere with the development of pathologies associated with diabetes mellitus.

## Author Contributions

BL, PS, and GJ were responsible for the study concept and design. BL, LP, ID, and PS were responsible for the data collection and analysis. BL, GJ, IN, and PS were interpreted the data, revised for intellectual content. All authors were involved in manuscript editing and have approved the final version for submission.

## Conflict of Interest Statement

The authors declare that the research was conducted in the absence of any commercial or financial relationships that could be construed as a potential conflict of interest.
